# Canagliflozin attenuates the progression of atherosclerosis and inflammation process in APOE knockout mice

**DOI:** 10.1186/s12933-018-0749-1

**Published:** 2018-07-26

**Authors:** Νarjes Nasiri-Ansari, Georgios K. Dimitriadis, Georgios Agrogiannis, Despoina Perrea, Ioannis D. Kostakis, Gregory Kaltsas, Athanasios G. Papavassiliou, Harpal S. Randeva, Eva Kassi

**Affiliations:** 1 Department of Biological Chemistry, National and Kapodistrian University of Athens Medical School, Athens, Greece; 2 Division of Translational and Experimental Medicine-Metabolic and Vascular Health, Warwick Medical School, University of Warwick, Coventry, CV4 7AL UK; 3 Division of Endocrinology and Experimental Medicine, Imperial College London, Hammersmith Campus, London, UK; 4 Human Metabolism Research Unit, WISDEM Centre, University Hospitals Coventry and Warwickshire NHS Trust, Coventry, CV2 2DX UK; 5 Laboratory of Pathological Anatomy, Medical School, National and Kapodistrian University of Athens, Athens, Greece; 6 Laboratory for Experimental Surgery and Surgical Research “N.S. Christeas”, Medical School, National and Kapodistrian University of Athens, Athens, Greece; 7 Second Department of Propedeutic Surgery, National and Kapodistrian University of Athens, Medical School, ‘Laiko’ General Hospital, Athens, Greece; 8 Division of Life and Health Sciences, Aston University, Birmingham, B4 7ET UK; 9 First Department of Internal Medicine, Laiko Hospital, National and Kapodistrian University of Athens, Athens, Greece

**Keywords:** Canagliflozin, SGLT2i, Atherosclerosis, Inflammation, APOE knockout mice

## Abstract

**Background:**

Sodium glucose co-transporter2 inhibitors reduce the incidence of cardiovascular events in patients with type 2 diabetes mellitus based on the results of recent cardiovascular outcome studies. Herein, we investigated the effects of long-term treatment with canagliflozin on biochemical and immunohistochemical markers related to atherosclerosis and atherosclerosis development in the aorta of apolipoprotein E knockout (Apo-E^(−/−)^) mice.

**Methods:**

At the age of 5 weeks, mice were switched from normal to a high-fat diet. After 5 weeks, Apo-E^(−/−)^ mice were divided into control-group (6 mice) treated with 0.5% hydroxypropyl methylcellulose and Cana-group (7 mice) treated with canagliflozin (10 mg/kg per day) per os. After 5 weeks of intervention, animals were sacrificed, and heart and aorta were removed. Sections stained with hematoxylin–eosin (H&E) were used for histomorphometry whereas Masson’s stained tissues were used to quantify the collagen content. Immunohistochemistry to assess MCP-1, CD68, a-smooth muscle actin, MMP-2, MMP-9, TIMP-1 and TIMP-2 expression was carried out and q-PCR experiments were performed to quantify mRNA expression.

**Results:**

Canagliflozin-group mice had lower total-cholesterol, triglycerides and glucose levels (*P *< 0.01), while heart rate was significantly lower (P < 0.05). Histomorphometry revealed that one in seven Cana-group mice versus four in six control mice developed atheromatosis, while aortic root plaque was significantly less, and collagen was 1.6 times more intense in canagliflozin-group suggesting increased plaque stability. Immunohistochemistry revealed that MCP-1 was significantly less expressed (*P *< 0.05) in the aortic root of canagliflozin-group while reduced expression of a-actin and CD68 was not reaching significance (*P *= 0.15). VCAM-1 and MCP-1 mRNA levels were lower (*P *= 0.02 and *P* = 0.07, respectively), while TIMP-1/MMP-2 ratio expression was higher in canagliflozin-group approaching statistical significance (*P *= 0.07).

**Conclusions:**

Canagliflozin attenuates the progression of atherosclerosis, reducing (1) hyperlipidemia and hyperglycemia, and (2) inflammatory process, by lowering the expression of inflammatory molecules such as MCP-1 and VCAM-1. Moreover, canagliflozin was found to increase the atherosclerotic plaque stability via increasing TIMP-1/MMP-2 ratio expression.

**Electronic supplementary material:**

The online version of this article (10.1186/s12933-018-0749-1) contains supplementary material, which is available to authorized users.

## Background

According to data from the World Health Organization (WHO), over 3 million people die worldwide from diabetes and its related complications every year, mainly due to cardiovascular disease (CVD) [1]. Despite paucity of information regarding the aetiopathogenesis of T2DM related cardiovascular complications, the toxicity of high blood glucose to the endothelium and other cells of the vessels seem to play an important role in the development of atherosclerosis and subsequent CVD. Atherosclerosis represents a systemic inflammatory process which implicates both cells of immune system and those of vessel wall. The basic pathologic lesion is atheromatous plaque. The atherogenic process evolves in different stages, starting from the endothelium activation/dysfunction and resulting in plaque vulnerability and rupture [2]. At the earlier stages of the atheromatous process, endothelial dysfunction/activation is characterized among others by increased expression of adhesion molecules and inflammatory molecules such as VCAM-1, ICAM-1 and MCP-1 and IL-6 by the endothelial and vascular smooth muscle cells. During the later stages of plaque rupture and/or erosion, among other factors, the metalloproteinases MMP-2, MMP-9 as well as their inhibitors TIMP-1 and TIMP-2; both expressed in endothelial cells and vascular smooth muscle cells, seem to play a critical role, since they regulate the collagen degradation of the extracellular matrix (ECM) [3]. Monocyte chemoattractant protein-1 (MCP-1) has been postulated to be a direct mediator of plaque instability [4].

SGLT2 inhibitors (SGLT2i) are a new class of oral anti-diabetic drugs, targeting the sodium-glucose co-transporter 2 which is the main glucose transporter of the kidney, and is responsible for reabsorption of 90% of glucose from primary urine. SGLT2 inhibition reduces the reabsorption of glucose and therefore enhances urinary glucose excretion, consequently decreasing both fasting and postprandial hyperglycemia and preventing glucotoxicity, and consequently hyperglycemia-induced damage. However, pleiotropic effects of these agents have already emerged [5].

Recent clinical trials amongst them CVD-REAL Nordi, EMPA-REG OUTCOME and canagliflozin CANVAS program have shown that SGLT2 inhibitors (dapagliflozin, empagliflozin, canagliflozin) use is associated with reduced cardiovascular disease and cardiovascular mortality compared with use of other glucose-lowering drugs in patients with T2DM, even though hemoglobin A1c (HbA1c) difference between randomized groups was marginal [6–8]. This suggests direct beneficial effects of SGLT2i on CVD risk besides the indirect effects attributed to better glycemic control, blood pressure or actions on extra-cardiovascular tissues such as adipose tissue.

Interestingly, recent studies have shown that SGLT-2 inhibitors can reduce pro-inflammatory IL-6, MCP-1 and ICAM-1 expression in blood vessels of rodent diabetic models, yet the molecular mechanisms remain largely unknown. To this direction, Mancini and collaborators reported recently that canagliflozin, but not empagliflozin or dapagliflozin can activate AMPK and inhibit IL-1β-stimulated secretion of IL-6 and monocyte chemoattractant protein-1 (MCP-1) in cultured human endothelial cells, while AMPK-independent mechanisms were also recognized [9]. Another research group has currently investigated the anti-inflammatory effects of SGLT-2 inhibitors in immune cells such as macrophages/monocytes both involved in the atherogenic process. According to their results, canagliflozin at clinically-relevant concentrations exerted the most potent inhibition-compared to other SGLT-2 inhibitors-of production and release of inflammatory factors IL1a, IL-6 and TNF-α [10]. These effects mediated by inhibiting intracellular glycolysis, enhancing autophagy, and promoting p62-mediated IL-1 degradation. Of note, enhanced autophagy and p62 levels might be mediated by increasing AMPK and NFκB activities, respectively; whether the above anti-inflammatory effects of canagliflozin were associated with SGLT2 should be further investigated [10].

Paradoxically, today there are more clinical than experimental data regarding the beneficial effects of SGLT2i on CVD, evaluating endothelial function, arterial stiffness, atherogenic cholesterols in patients with DM type 2 etc. [11–13]; however, although various SGLT2i such as empagliflozin, dapagliflozin, ipragliflozin and luseogliflozin have been evaluated in animal experimental studies regarding their the anti-atherogenic effects, there is paucity of evidence regarding canagliflozin.

To this context, we investigated for the first time the effects of long-term treatment with canagliflozin on atherosclerosis development in the aorta of APOE^(−/−)^ mice as well as on biochemical and immunohistochemical markers linked to atherosclerosis.

## Materials and methods

### Animals

APOE^(−/−)^ mice (on the genetic background C57BL/6) were originally purchased from “The Jackson Laboratory” and bred in the animal facility of National and Kapodistrian University of Athens. Mice were kept at specific pathogen free (SPF) controlled environment (22–26 °C temperature, 40–60% humidity and 12 h light/dark cycle).

#### Experimental protocols

Thirteen male APOE^−/−^ mice were kept on a standard rodent chow. At the age of 5 weeks mice were switched to high fat diet (20–23% by weight; 40–45% kcal from fat) containing cholesterol (0.2% total).

After 5 weeks, mice were randomly divided into two groups (1) canagliflozin-group 10 mg/kg/day (n = 7) administered orally by gavage, and (2) control-group (n = 6) administered the same volume of 0.5% hydroxypropyl methylcellulose/day (vehicle), via gavage. After 5 weeks of oral treatment with canagliflozin or vehicle, mice were sacrificed under isoflurane anesthesia by transection of the diaphragm and, the aorta along with heart were rapidly excised. Food intake and body weight were measured once weekly over a period of 10 weeks. Blood glucose levels were also measured after 8–10 h fast via tail puncture at baseline, before canagliflozin/vehicle oral administration, once during experiment (3 week) and before experiment endpoint. Canagliflozin was purchased from Selleck Chemand dissolved in 0.5% hydroxypropyl methylcellulose.

### Mice blood pressure measurement

Blood pressure was measured once at baseline, before canagliflozin oral administration began and once before sacrificing animals. Mice were acclimatized to the restrainer on a warming pad for 2 consecutive days before final measurements. Measurements were performed in quiet environment to avoid causing mice anxiety. Blood pressure measurements were performed (15–25 measurements/mouse) using a computerized non-invasive tail-cuff system (CODAs, Kent Scientific, USA). All measurements are expressed as mean value of heart rate, systolic and diastolic blood pressure.

### Serum analysis of biochemical parameters

Blood was drawn once before the onset of canagliflozin administration from the facial vein and once by heart puncturing after sacrificing mice. Serum glucose, cholesterol, triglycerides, and HDL- and LDL-cholesterol levels were determined using a dedicated autoanalyzer.

### RNA isolation and real time PCR

Total RNA was extracted from fresh frozen aorta using RNeasy kit (Qiagen). Extracted mRNA was then reverse transcribed into cDNA using the iScript cDNA synthesis kit (Bio-Rad). Real-time PCR analysis was performed as described previously [14]. The expression of Matrix Metalloproteinase (MMP-2 and MMP-9) and their inhibitors (TIMP-1 and TIMP-2), IL-6, intercellular adhesion molecule 1 (ICAM-1), vascular cell adhesion molecule 1 (VCAM-1) and monocyte chemotaxis protein (MCP-1) was measured using Luna^®^ Universal qPCR Master Mix (New England Biolabs) on a CFX96 (Bio-RAD). The sequences of primers used for RT-PCR analysis in this study are listed in Table 1. A melting curve analysis was performed to confirm the specificity of qPCR products. Fold-changes were calculated using the 2^−∆∆Ct^ method and were normalized against 18s rRNA expression. All reactions were performed in triplicates and repeated three times.

**Table 1:** qPCR primer sequences used in this study

Target gene	Primer sequence (forward)	Primer sequence (reverse)
MMP-2	5^′^-CCCTCAAGAAGATGCAGAAGTTC-3^′^	5^′^-TCTTGGCTTCCGCATGGT-3^′^
MMP-9	5^′^-CGTCGTGATCCCCACTTACT-3^′^	5^′^-AACACACAGGGTTTGCCTTC-3^′^
TIMP-1	5^′^-GCATGGACATTTATTCTCCACTGT-3^′^	5^′^-TCTCTAGGAGCCCCGATCTG-3^′^
TIMP-2	5^′^-TTCCGGGAATGACATCTATGG-3^′^	5^′^-GGGCCGTGTAGATAAACTCGAT-3^′^
MCP-1	5^′^-GCATCCACGTGTTGGCTCA-3^′^	5^′^-CTCCAGCCTACTCATTGGGATCA-3^′^
IL-6	5′-CCTCTGGTCTTCTGGAGTACC-3′	5′-ACTCCTTCTGTGACTCCAGC-3′
ICAM-1	5^′^-GGTTCTCTGCTCCTCCACAT-3^′^	5^′^-CCTTCCAGGCTTTCTCTTTG-3^′^
VCAM-1	5^′^-CTTCCAGAACCCTTCTCAG-3^′^	5^′^-GGGACCATTCCAGTCACACTTC-3^′^
18S rRNA	5^′^-GTTCCGACCATAAACGATGCC-3^′^	5^′^-TGGTGGTTGCCCTTCCGTCAAT-3^′^

### Histochemistry and immunohistochemistry

#### Quantification of atherosclerotic lesion area

Aortic tissues were fixed and embedded in paraffin. The 4-μm-thick sections were stained with hematoxylin–eosin (H&E) and used for histopathological analysis whereas Masson’s trichrome stained sections were used to quantify tissue section’s collagen content. The degree of pathological changes was evaluated microscopically by measuring the area of atheromatous plaques. Results are reported as the percentage of the neointima area containing the lesion. Threshold was set and the positively stained area for each histochemical stain was automatically calculated and then the percent of the positively stained area to the total cross-sectional vessel wall area or intimal plaque lesion area was reported. Plaque area analysis was carried out using Image Pro Plus software version 5.1 (Media Cybernetics, Inc.).

#### Immunohistochemistry

For immunohistochemistry, all sections were deparaffinized at 60 °C. Antigen retrieval was performed using citrate buffer (PH.6.0) for 7 min at 100 °C followed by blocking with normal goat serum (CST, 5425S) for 1 h. Slides were then incubated with appropriate concentration of primary antibodies against CD68 (ZYTOMED, MSK055), α-smooth muscle actin (ZYTOMED, MSK030), MCP-1 (ACRIS, AM32136PU-N), MMP-2 (Proteintech Group, 103732-AP), MMP-9 (Proteintech Group, 10375-2-AP) and their inhibitors TIMP-1 (Santa Cruz Biotechnology, *sc*-*21734*) and TIMP-2 (Santa Cruz Biotechnology, sc-21735) followed by incubation with corresponding secondary antibody conjugated to horseradish peroxidase (ZYTOMED, ZUC053-100) and visualized by applying DAB (CST.8059P). All slides were counterstained with hematoxylin and integral absorbance was examined under light microscope and results were quantified using Image Pro Plus software version 5.1 (Media Cybernetics, Inc.). A positive tissue control was used to ensure the specificity of antibodies used in this study.

### Statistical analysis

Normality of quantitative data distribution was assessed using Shapiro–Wilk test. Student’s t-test, Welch’s t-test or Mann–Whitney U test were used for comparisons between two groups with quantitative data as appropriate. Chi square test or Fisher’s exact test were used for comparisons among groups with qualitative data as appropriate. Correlations between quantitative parameters were tested with Pearson’s correlation coefficient or Spearman’s rank correlation coefficient as appropriate. All tests were two-tailed, and results were considered statistically significant if the *P*-value was < 0.05. Statistical analysis was performed using the 23rd edition of statistical package for social sciences (SPSS) (IBM Corporation, Armonk, NY, USA).

## Results

### Oral administration of canagliflozin for 5 weeks improved heart rate and biochemical/metabolic parameters associated with atherosclerosis

No significant difference in daily food intake was observed between the two groups. Nevertheless, body weight was significantly increased in both groups following HFD and 5 weeks of oral canagliflozin/vehicle administration as compared to the value measured at experiment baseline. No significant difference in weight gain was observed between canagliflozin and control group (Additional file 1: Figure S1).

**Fig. 1:**
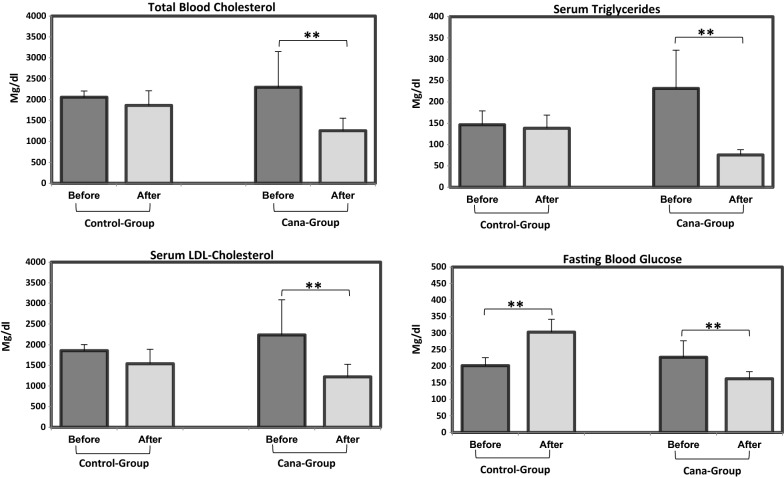
Serum lipid and fasting blood glucose levels in Cana- and control-groups after 5 weeks of canagliflozin/vehicle oral administration. Significant reduction in total cholesterol, triglyceride, LDL-cholesterol and fasting blood glucose levels was observed in Cana-group at the end of experimental procedure compared to baseline. Fasting glucose was the only significantly increased parameter observed in the control group at the end of intervention. Data are shown as mean ± SD (***P ≤ 0.001, **P ≤ 0.01)

Fasting blood glucose (8 h of fasting) and serum lipid levels were measured before canagliflozin/vehicle oral administration as well as at the end of intervention period. A significant reduction in glucose, total cholesterol, LDL-cholesterol and triglyceride levels (*P *< 0.01) were observed in canagliflozin group (Fig. 1). After treatment with canagliflozin, glucose levels returned to normal range, contrary to placebo group where glucose increased significantly above normal range with glucose level progression to diabetes range (*P* < 0.001).

At the end of intervention period, total cholesterol, glucose and triglyceride levels were significantly lower in Cana-group (*P *= 0.01, *P *= 0.001, *P *= 0.02 respectively). Moreover, diastolic blood pressure values was significantly higher in the control group at experiment endpoint (*P *= 0.05) (Additional file 2: Table S1).

**Fig. 2:**
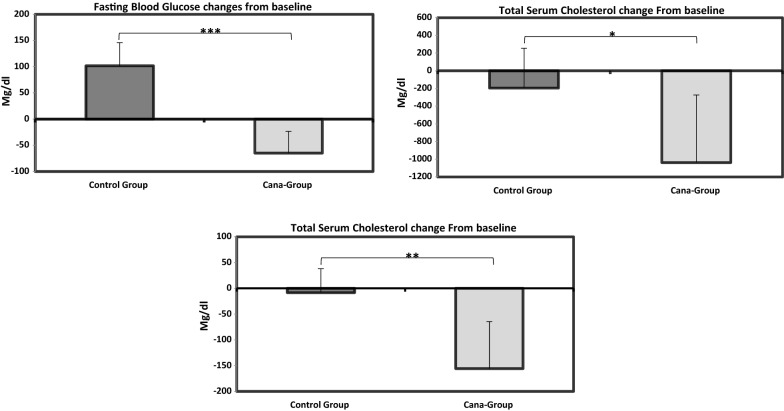
Fasting glucose, total cholesterol and triglyceride changes from baseline. Significant changes were detected from baseline in fasting glucose, total cholesterol and triglyceride between study groups. Data are shown as mean ± SD (***P < 0.001, **P < 0.01, *P < 0.05)

At the end of canagliflozin/placebo oral treatment, there was a significant difference from baseline in fasting glucose (*P *< 0.001), triglycerides (*P *< 0.01), and total cholesterol (*P *< 0.05) between groups. Mean ± SD changes in LDL-, HDL-cholesterol and creatinine levels from baseline were similar in both groups (Fig. 2).

**Fig. 3:**
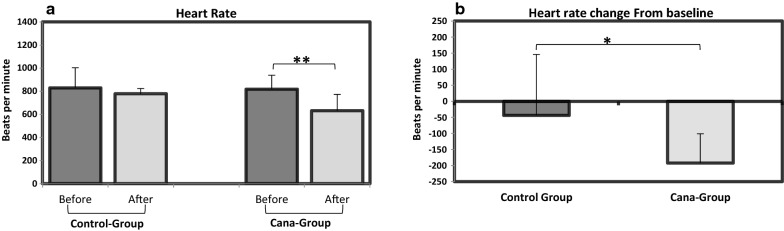
Canagliflozin/vehicle oral administration effect on heart rate of APOE^(−/−)^ mice. **a** 5 weeks of canagliflozin intervention led to significant reduction of heart rate (**P < 0.01) while no significant difference was observed in the control group. **b** Heart rate changes from baseline were significantly different between Cana- and control-groups (*P < 0.05)

Canagliflozin significantly reduced heart rate (**P ≤0.01) (Fig. 3), whereas no significant change was observed in the control group (Fig. 3a). This finding was confirmed by comparing heart rate changes from baseline (value measured before onset of canagliflozin/vehicle oral administration) between the two groups. (*P ≤ 0.05) (Fig. 3b). As it is shown in Additional file 2: Table S1 post-treatment heart rate was reduced in Cana-group compared to control-group approaching statistical significance (*P *= 0.076).

### Canagliflozin reduces atherosclerosis lesion formation and increases collagen content

**Fig. 4:**
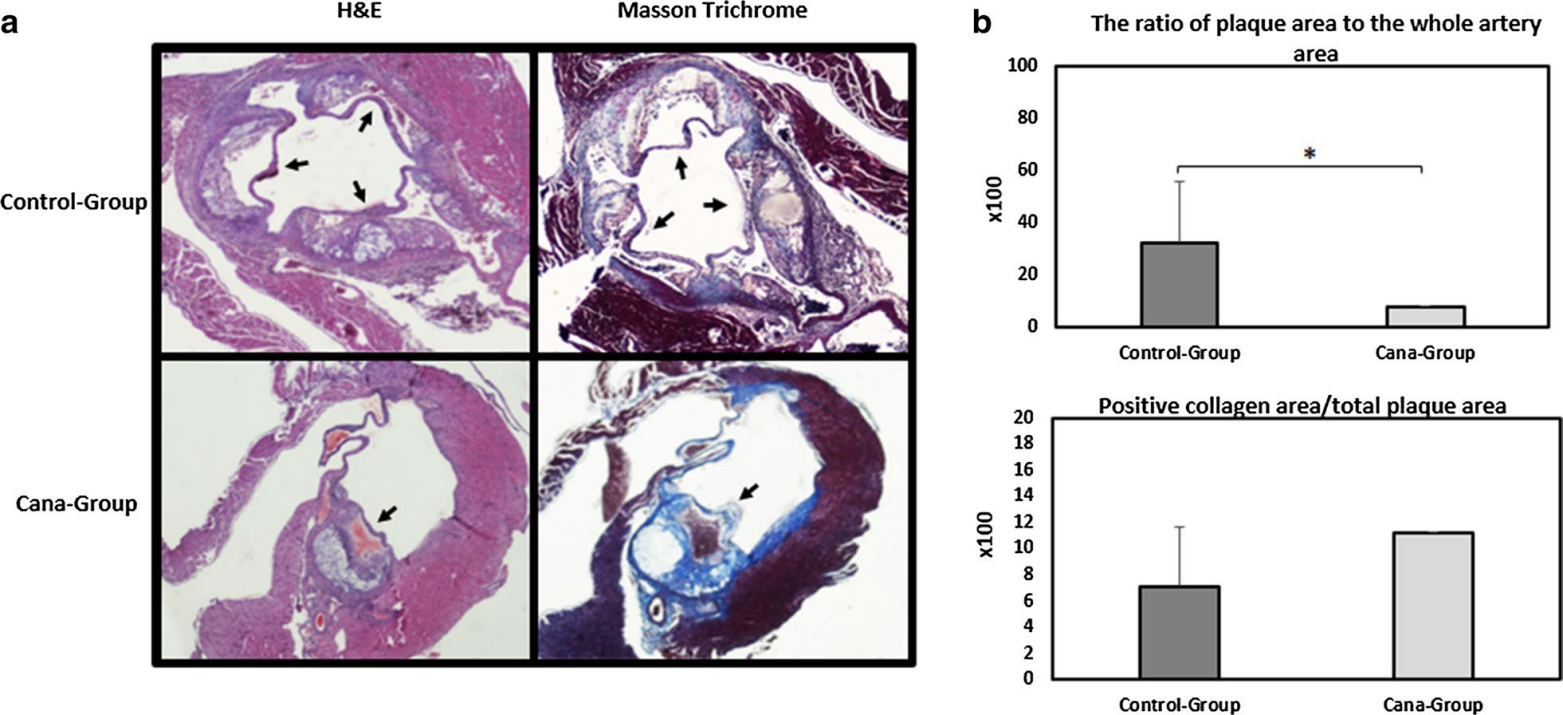
Atherosclerotic plaque extension among APOE^(−/−)^ mice on western diet treated with canagliflozin (Cana-group) or vehicle (control-group). **a** Selected 4 μm section images from the aortic root stained with H&E and Masson trichrome. Formation of atherosclerotic plaque was examined using H&E staining while histological examination of atherosclerotic plaque collagen content was assessed using Masson trichrome staining. **b** Quantification of plaque area is shown as a percentage of lumina stenosis by thickened intima. Collagen content was measured using quantification of Masson trichrome positive area over complete plaque area. Values are shown as mean ± SD and *P < 0.05. Original magnification ×40. H&E indicates hematoxylin and eosin

Canagliflozin administration for 5 weeks significantly reduced atherogenesis process. One mouse in the Cana-group (7 mice) developed atherosclerotic plaque, contrary to four mice with atherosclerotic plaque in the control-group (6 mice). Atherosclerotic plaque presence was assessed using H&E staining (representative Fig. 4a). Atherosclerotic lesion area was quantified measuring the percentage of lumen area covered by total plaque area in all aortic root sections, and the mean plaque area (± SD) was then calculated for each group (Fig. 4b). The formation of atherosclerosis was significantly lower (by 25%) in Cana-group (*P *< 0.05). Masson Trichrome staining showed that atherosclerotic lesions in the Cana-group had greater collagen content (1.6 times more) compared to placebo group (*P *< 0.1). Images and quantitative data are shown in Fig. 4.

### Canagliflozin reduced the expression of inflammatory molecules and improved metalloproteinase profile

To evaluate the effect of canagliflozin treatment on the expression of inflammatory (IL-6, MCP-1), adhesion molecules (ICAM-1, V-CAM-1), matrix metalloproteinases (MMP-2, MMP-9) and their inhibitors (TIMP-1, TIMP-2), total RNAs were isolated from the thoracic aorta and analyzed using real-time quantitative RT-PCR.

**Fig. 5:**
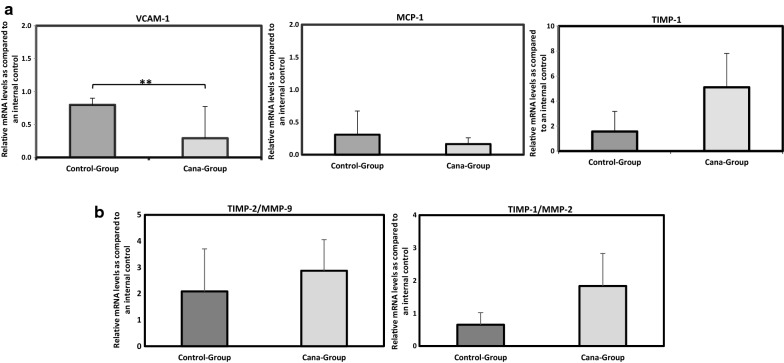
**a** VCAM-1, MCP-1 and TIMP-1 mRNA expression from thoracic aorta of mice treated with canagliflozin (10 mg/kg/day—5 weeks) and control-group (vehicle—5 weeks). VCAM-1 mRNA expression was significantly decreased in Cana-group as well as MCP-1, while TIMP-1 mRNA expression was increased. **b** TIMP-1/MMP-2 ratio mRNA was also increased in Cana-group (compared to control-group) approaching the borderline of significance (P = 0.07). Data are presented as mean (**P < 0.01)

**Fig. 6:**
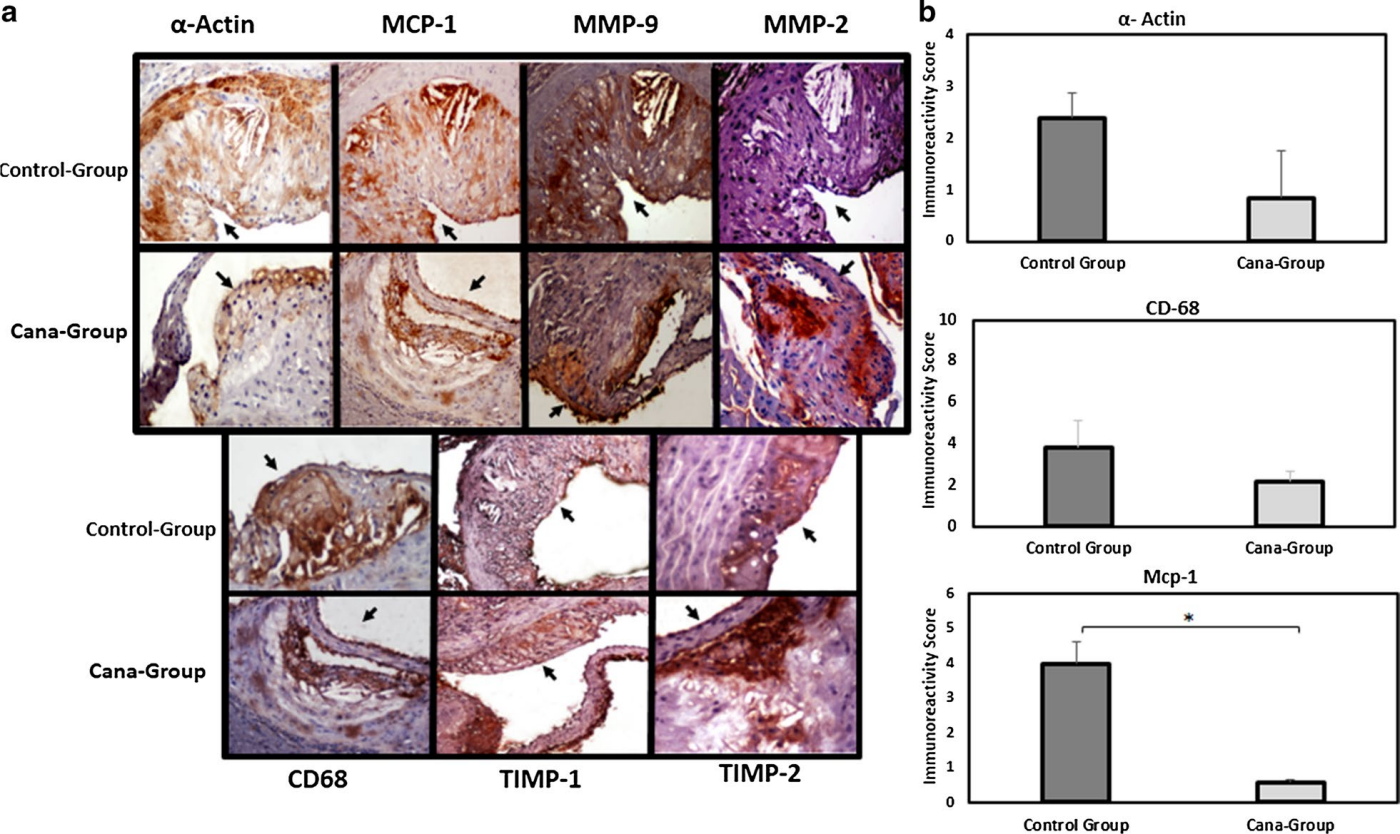
Atherosclerotic lesion characteristics in APOE^(−/−)^ mice fed with HFD and treated with canagliflozin (Cana-group) or vehicle (control-group). **a** Selected 4 μm image sections from the aortic root, immunostained for smooth muscle cells (α-Actin), MCP-1, MMP-9 and MMP-2, macrophages (CD68), and TIMP-1/TIMP-2. **b** Immunostaining differences between groups. Positive cell proportion stained with each antibody was scored from 0 to 4 and staining intensity from 0 to 3 with 0 representing no visual staining and 4 representing intense staining (> 75% of area). Immunoreactivity was scored on a 0–7 scale [extend of staining (0–4) plus intensity of staining (0–3)]. There was a significant difference in MCP-1 expression between Cana-group and control-group (P = 0.048). A-actin and CD68 expression were numerically higher in the Control-group (P = 0.1). Bar graphs show mean ± SD and *P < 0.05. (Original magnification ×200; scale bar, 150 μm)

We demonstrate that oral canagliflozin administration significantly reduces VCAM-1 mRNA levels (*P *= 0.01) while marginally induces TIMP-1 and decreases MCP-1 mRNA expression levels (*P *= 0.07). Canagliflozin treatment causes no significant alteration in IL-6, ICAM-1, MMP-2, MMP-9 and TIMP-2 mRNA levels compared to controls (Fig. 5a). A balance between MMPs and TIMPs is known as an indicator of MMPS overall collagenolytic activity. To this end, TIMP-1/MMP-2 ratio mRNA levels was measured. Our findings demonstrate that TIMP-1/MMP-2 ratio mRNA level was higher in Cana-group (Fig. 5b), while approaching significance (*P *= 0.07). Aortic root section immunohistochemistry revealed that smooth muscle (α-actin) and macrophage (CD68) cells content from atherosclerotic plaques were marginally higher in the control group (*P *< 0.1). Moreover, treatment with canagliflozin led to significant reduction in MCP-1 expression (*P *< 0.05) and a marginal increase in atherosclerotic plaque TIMP-1 expression (*P *< 0.1). Images and quantitative data are shown in Fig. 6.

## Discussion

Canagliflozin is an intermediate-acting SGLT2i with proven clinical efficacy regarding glycemic control, blood pressure and weight reduction, in patients with T2DM irrespective of the degree of CVD history or risk factors [15]. In the present study, we investigated the effect of canagliflozin on atherosclerosis formation and demonstrated for the first time, that 5 week canagliflozin administration attenuates atheromatous process in APOE^(−/−)^ mice fed an atherogenic diet for 10 weeks enough for atheroma to be formed [16].

We evaluated the impact of treatment with canagliflozin in established atherosclerosis risk factors and were able to demonstrate the anticipated effect on fasting glucose level. In fact, the control-group fed with western diet for 10 weeks, had significantly increased fasting blood glucose level while canagliflozin administration reversed this effect. In contrast, Terasaki et al. [17] found that western diet fed mice for 4 weeks retained their glucose levels in the normal range, an effect that is probably attributed to strain background differences [18]. It should be noted that the majority of available animal data on glucose-lowering effects of SGLT2 inhibitors have used models of streptozotocin-induced diabetic models.

Canagliflozin administration combined with atherogenic diet, did not lead to reduced weight. On the contrary, there was a statistically significant increase, without differences between the two groups either in weight or in daily food intake. Although weight-loss effects of canagliflozin have been demonstrated in clinic trials, with reduction in both subcutaneous and visceral adipose tissue dose-dependently [19, 20], animal studies have yielded conflicting results. Administration of canagliflozin 30 mg/kg/day for 4 weeks reduced the weight gain in diet-induced obese mice fed with high-fat diet [21]. In a recent study by Ji et al. [22] administration of 60 mg/kg/day canagliflozin for 4 weeks in mice fed with high-fat diet containing 6% fat, reduced significantly the body weight, via reduction of PPRAγ in the liver; interestingly, in line with our results, this effect was not achieved with the lower dose of 15 mg/kg/day. Naznin et al. [23] found that canagliflozin at dose of 30 mg/day attenuated the body weight gain by promoting caloric loss and suppression of obesity-related inflammation in both the nervous system and skeletal muscle. Regarding the effect of other SGLT2 is on body weight, administration of dapagliflozin for 12 weeks in non-diabetic APOE^(−/−)^ mice did not significantly reduce weight [24]. In another study, dapagliflozin did not change the weight of APOE^(−/−)^ mice, with or without diabetes [17] while ipragliflozin 1 mg/kg/day for 4 weeks, reduced significantly the weight of db/db rats [17]. Empagliflozin for 8 weeks decreased body weight and particularly adipose tissue in APOE^(−/−)^ mice, while did not affect the weight of ZDF rats (type 2 diabetes model) [25]. Among the involved mechanisms, decrease of the subcutaneous fat mass and the size of visceral adipocytes as well as enhanced fat utilization and browning, attenuation of obesity-induced inflammation and insulin resistance by polarizing M2 macrophages in WAT and liver have been described [26, 27]. Although, the daily food intake and body weight changes did not differ between our two groups, canagliflozin exerted beneficial effects on lipid profile, reducing total cholesterol and triglycerides. As with body weight, there are also inconsistent results concerning the effects of SGLT-2 inhibitors on lipids, either reducing or not affecting total cholesterol, LDL-cholesterol and triglycerides, however data examining the effects specifically of this dose of canagliflozin on lipid profile have not been assessed previously [22, 24, 25, 28–30]. Conflicting results concerning the effects of SGLT-2i on body weight and lipid profile may be due to differences in animal models used (i.e. mice, rats, diabetic, non-diabetic, atherosclerotic, obese etc.), different drugs used (including different doses and durations of treatment), and/or different diets.

Although clinical studies have proved the antihypertensive effects (SBP, DBP, pulse pressure and mean arterial pressure) of canagliflozin [12], herein we find just a borderline reduction of diastolic pressure. However, canagliflozin reduces significantly heart rate. This could be attributed to a possible decrease in insulin levels as a result of the glucose-lowering effects of canagliflozin since it is well known that insulin increases sympathetic activity [31]. Although we did not measure insulin levels, reduction in serum insulin has been reported following administration of empagliflozin for 7 weeks in a metabolic syndrome model rat [26]. It should be mentioned that Terasaki et al. [17], showed no differences in heart rate following administration of dapagliflozin for 4 weeks, however their APOE^(−/−)^ mouse model feeding western diet did not increase blood glucose levels, thus there were no changes in blood glucose with dapagliflozin administration.

It is well established that inflammatory cytokines and adhesion molecules play a crucial role in the initiation and progression of atherosclerotic process. Since we found that the majority of the mice in Cana-group did not form atherosclerotic plaque in contrast to control-group, we investigated the expression of ICAM-1, VCAM-1, IL-6 and MCP-1 between groups and found lower mRNA expression of MCP-1 and VCAM-1. A study by Oelze et al. [32] showed that empagliflozin for 6 weeks decreased the expression of IL-6 and MCP-1. Moreover, luseogliflozin also reduced the expression of ICAM-1 and IL-6 while did not affect VCAM-1 [30]. In both studies, streptozotocin-induced diabetes models were used.

Interestingly, we additionally confirmed the beneficial effect of canagliflozin on MCP-1 expression at the protein level, in atherosclerotic lesion (plaque). The role of MCP-1 in both initiation and progression of atherosclerosis has been well-characterized and various mechanisms have been proposed for this [33]. A potential mechanism is by promoting the recruitment of monocytes/macrophages in atherosclerotic lesion. In our study, the decreased number of stained macrophages in the plaque could be attributed to, among others, decreased MCP-1 expression. Moreover, it has been reported that MCP-1 induces MMP-2 expression in human endothelial cells, as well as the expression of MMP-9 in human smooth muscle cells [34, 35]. Both MMPs are critical factors involved in plaque destabilization, through degradation of collagen-rich extracellular matrix.

According to our data, although canagliflozin administration reduced MCP-1 expression, it did not change the MMP-2, MMP-9, while marginally increased TIMP-1 and TIMP-1/MMP-2 ratio, indicating reduced activity of MMP-2.

Previous studies have shown that high glucose concentrations decrease the expression of MMPs and increase the expression of their inhibitors (TIMPs) [36]. In our study, by reducing blood glucose at normal levels, canagliflozin would be expected to lead to the above-mentioned profile (increased MMP-2 and MMP-9 expression and decreased TIMP-1 and TIMP-2). However, acting via decreasing either directly, or indirectly-through lowering blood glucose [37], MCP-1, not only counterbalances but rather is associated with a favorable TIMP-1/MMP-2 profile in the aortic lesion. Another potential mechanism that could explain this TIMP-1/MMP-2 profile is the reduction of VCAM-1 by canagliflozin which has been demonstrated to lead to an up-regulation of TIMP-1 [38]. This favorable profile could result, along with other factors, in increased collagen content demonstrated in the plaque of the Cana-group. Of note, another SGLT-2 inhibitor, luseogliflozin, given for 1 week decreased MMP-2 and MMP-9 expression in aorta wall but not in atherosclerotic plaque of streptozotocin-induced diabetic APOE^(−/−)^ mice [30].

Attenuation of plaque formation and decreased number of invasive macrophages has been demonstrated following dapagliflozin administration in streptozotocin-induced APOE^(−/−)^ mice, while no effect has been reported in non-diabetic APOE^(−/−)^ mice [17]. Moreover, empagliflozin administration for 8 weeks decreased the burden of plaque (plaque area), expression of inflammatory molecules TNF, IL-6 and MCP-1, and invasion of plaque by inflammatory cells and this effect was more potent in the empagliflozin mice group compared to glimepiride-group that achieved the same glycemic control, the latter suggesting beneficial effects of the SGLT-2i other than just improved glycemic control [25].

According to our data, increased staining for α-actin (smooth muscle cells) in the control-group (4 mice) compared to Cana-group (one mouse with plaque)-albeit not statistically significant-could suggest decreased plaque stability in Cana-group, an effect contradicting our collagen content findings, requiring further clarification. However, recent studies propose, a heterogeneous population of cells expressing α-actin and 50% of foam cells within advanced human coronary artery lesions. These, express α-actin besides CD68 (macrophage marker), whereas ≤ 80% of the lesion cells (including mesenchymal stem cells and macrophage-like cells) are smooth muscle cell-derived [39, 40]. An important advantage of this study is that we used a mouse model that (1) can develop all stages of atherosclerosis process, from endothelium activation and foam cell stage to plaque vulnerability and rupture, and (2) can develop mild diet-induced diabetes, thus is a more suitable model for studying diabetes and its main complication i.e. atherosclerosis. It should be noted that streptozotocin-induced diabetic mice (resembling type 1 diabetes), that are used in most studies elucidating the effects of other SGLT-2 inhibitors, are characterized by markedly increased glucose levels which result in more detrimental effects on various inflammatory and metabolic parameters contributing to atherosclerosis, thus their beneficial effects could be more pronounced. Our results showing mitigation of atherosclerosis in mice with mild diet-induced diabetes, are of great importance. Moreover, two time-points (before and after the intervention) measurement of biochemical parameters, gave us the chance to compare their changes and not only the values at the end of intervention, between the two groups.

Limitations of our study is the small number of mice and the fact that the design of this study does not allow to draw conclusions around the possible direct effects of canagliflozin on atherosclerotic process. Furthermore, measurements of serum insulin levels as well as of visceral fat could add substantially to the elucidation of the mechanism via which canagliflozin can reduce atheroma burden. Finally, although the significant difference in the development of atherosclerosis between our two groups substantiates the anti-atherogenic effect of canagliflozin, can at the same time make the interpretation of data regarding plaque stability difficult.

Attenuation/inhibition of atherosclerosis in our model is mainly attributed to the glucose and lipid-lowering effects of canagliflozin. Correlation analysis showed that the atherosclerotic area is related to glucose and LDL-cholesterol range after the intervention; however, direct effects of canagliflozin could not be ruled out especially in the light of recent studies demonstrating direct effects of canagliflozin on human endothelial cells and monocyte/macrophages, both involved in atherogenesis process [9, 10]. Of note, SGLT2 is not detected at mRNA level in human endothelial cells, while it remains uncertain if SGLT2 protein is present [9]. Thus, whether the above anti-inflammatory effects of canagliflozin are associated with SGLT2 or SGLT1 which is expressed in endothelial cells [41], or another facilitative glucose transporter-as it is suggested previously in rat muscle cells-remains unexplored and of great interest [21].

In summary, our data provide for the first time, evidence that canagliflozin attenuates atherosclerosis process in atherosclerotic mouse model through mechanisms that involve (1) improved glycemic control and decreased cholesterol and triglycerides, and (2) inflammation process via decreasing the MCP-1 and VCAM-1 expression. Moreover, canagliflozin seems to increase the stability of atherosclerotic plaque and possible mechanisms involve decreased MCP-1 expression and increased TIMP-1/MMP-2 ratio. Further experimental studies with larger number of mice per group based on power calculation, (including a group of atherosclerotic mice model that do not become diabetic with atherogenic diet), longer duration as well as various doses, will add to current knowledge and importantly will delineate possible direct effects of canagliflozin on the atherosclerosis process. Elucidation of the precise molecular mechanisms underpinning SGLT2 signalling in cells involved in the atherogenic process may prove useful in understanding the role of canagliflozin in the CVD.

## Additional files

**Additional file 1: Figure S1.** Changes in food intake and weight between groups in response to treatment.

**Additional file 2: Table S1.** Biochemical parameters and vital signs at the end of five-week intervention in both groups.

## Abbreviations

SGLT2: sodium glucose co-transporter2
SGLT2i: sodium glucose co-transporter2 inhibitor
T2DM: type 2 diabetes mellitus
Apo-E^(−/−)^: apolipoprotein E knockout Apo-E^(−/−)^
CVD: cardiovascular disease
MCP-1: monocyte chemoattractant protein 1
CD68: cluster of differentiation 68
MMP-2: matrix metalloproteinase-2
MMP-9: matrix metalloproteinase-9
TIMP-1: tissue inhibitor of metalloproteinases-1
TIMP-2: tissue inhibitor of metalloproteinases-2
ICAM-1: intercellular adhesion molecule 1
VCAM-1: vascular cell adhesion molecule 1
Cana: canagliflozin
ECM: extracellular matrix
HbA1c: hemoglobin A1c
Il-6: intrleukin 6
HFD: high fat diet
DBP: diastolic blood pressure
SBP: systolic blood pressure
