# Supplementary figures and images for: Canagliflozin attenuates the progression of atherosclerosis and inflammation process in APOE knockout mice
Source: Cardiovasc Diabetol. 2018 Jul 26;17:106. doi: 10.1186/s12933-018-0749-1 (PMC6063004; doi:10.1186/s12933-018-0749-1)

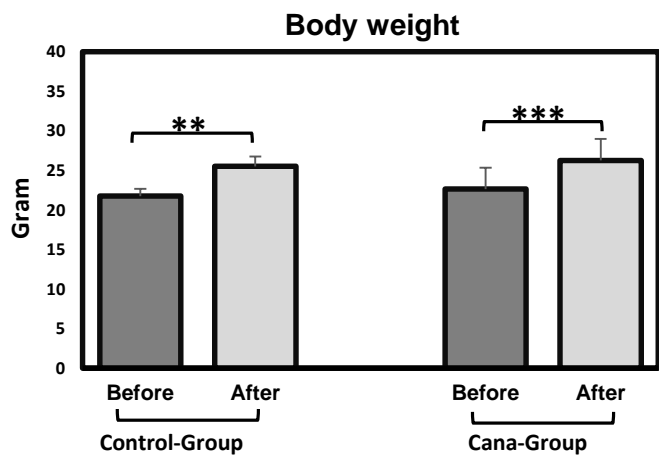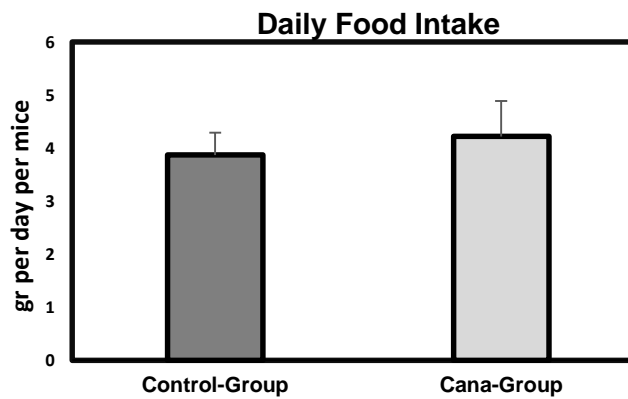

Supplement: Supplementary file 1 — Additional file 1: Figure S1. Changes in food intake and weight between groups in response to treatment. [file 12933_2018_749_MOESM1_ESM.pdf]
