# Supplementary material for: Canagliflozin attenuates the progression of atherosclerosis and inflammation process in APOE knockout mice
Source: Cardiovasc Diabetol. 2018 Jul 26;17:106. doi: 10.1186/s12933-018-0749-1 (PMC6063004; doi:10.1186/s12933-018-0749-1)
Supplement: Supplementary file 2 — Additional file 2: Table S1. Biochemical parameters and vital signs at the end of five-week intervention in both groups. [file 12933_2018_749_MOESM2_ESM.doc]

| **Serum Biochemistry** | | | |
| --- | --- | --- | --- |
|  | **Cana-Group (n=7)** | **Control-Group (n=6)** | **p-Value** |
| **Fasting Blood Glucose** | 162.14±21.69 | 303.33±38.59 | **0.00001** |
| **Total-Cholesterol** | 1259±299.06 | 1865±348.4 | **0.01** |
| **HDL-Cholesterol** | 85.5±15.95 | 112.67±24.01 | 0.061 |
| **LDL-Cholesterol** | 1224.43±281.72 | 1541.33±205.61 | 0.11 |
| **Triglycerides** | 75.83±12.39 | 138.3333±30.77 | **0.002** |
| **Creatinine** | 2.7±0.82 | 2.57±0.49 | 0.81 |
| **Heart Rate, Systolic & Diastolic Blood Pressure** | | | |
| **Heart Rate** | 631.5±141.43 | 778.08±46.43 | 0.076 |
| **Systolic Blood Pressure** | 90.43±10.4 | 101.566±10.12 | 0.12 |
| **Diastolic Blood Pressure** | 60.86±7.24 | 70.94±7.4 | **0.05** |

**Table S1.** Biochemical parameters and vital signs at the end of five-week intervention in both groups.

Data are shown as means ± SD and P- values < 0.05 were considered statistically significant*.*
